# Head and neck manifestations of extramedullary plasmacytomas and their differential diagnoses: a pictorial review

**DOI:** 10.1093/bjr/tqaf031

**Published:** 2025-02-11

**Authors:** Sophie Wilkinson, Susan Jawad, Xin-Ying Kowa

**Affiliations:** Department of Imaging, University College London Hospitals NHS Foundation Trust, London NW1 2BU, United Kingdom; University College London, Centre for Medical Imaging, Division of Medicine, London WC1E 6JF, United Kingdom; Department of Imaging, University College London Hospitals NHS Foundation Trust, London NW1 2BU, United Kingdom

**Keywords:** extramedullary plasmacytoma, multiple myeloma, head and neck manifestations of extramedullary plasmacytoma, solitary plasmacytoma

## Abstract

Solitary plasmacytoma and multiple myeloma are plasma cell dyscrasias that typically present as bone tumour(s) due to abnormal and unregulated proliferation of plasma cells in the skeletal marrow. Both can manifest as soft tissue lesions within the head and neck region, and they may pose a diagnostic challenge given their relative rarity. This review aims to highlight the key multimodality imaging features of plasma cell neoplasms with an emphasis on extraosseous/extramedullary presentations in the anatomically complex head and neck region, as well as the relevant anatomy of various head and neck subsites and the main differential diagnoses that the radiologist should consider. Familiarity with these entities is crucial and will help the radiologist facilitate the timely diagnosis of plasma cell neoplasia using the arsenal of increasingly available and sensitive imaging techniques.

## Introduction

A plasmacytoma is a tumour of abnormal plasma cells that can occur within bone or soft tissues.^1^ Solitary plasmacytoma (SP) and multiple myeloma (MM) are distinct types of plasma cell disorders that are characterized by the monoclonal proliferation of plasma cells and excessive production of the paraprotein M immunoglobulin.^1^ Despite being clinically defined as distinct plasma cell dyscrasias, both can present with extraosseous soft tissue lesions known as extramedullary plasmacytoma (EMP).

- Symptomatic MM is a mature B-cell neoplasm defined by the International Myeloma Working Group (IMWG) criteria as >10% of clonal plasma cells within bone marrow or biopsy-proven bony or EMP, and at least 1 myeloma-defining event. Myeloma-defining events are either traditional CRAB features (hypercalcemia, renal failure, anaemia, and/or lytic bone lesion) or biomarkers of malignancy.^2^ According to the updated IMWG definition, a myeloma-defining event now includes >1 focal lesion detected on magnetic resonance imaging (MRI), highlighting the importance of radiological detection.^3^
- When plasmacytomas occur in clinical isolation, they are known as SP. Solitary plasmacytoma is differentiated from MM by the absence of the abovementioned IMWG defined criteria.^2^

EMP are uncommon, accounting for 3-5% of the manifestations of all plasma cell dyscrasias, but with a high proportion (80%) occurring within the head and neck, it is an important differential diagnosis to consider.^4^^,^^5^

Plain film skeletal surveys have long been superseded by cross-sectional imaging techniques in the work-up of patients with suspected MM.^3^ The current recommendation includes whole-body imaging techniques utilising low-dose computed tomography (CT), MRI, or 18-FDG-PET-CT (18-fluorodeoxyglucose positron emission tomography-CT) depending on local guidelines alongside clinical and radiological findings.^3^ Whole-body MRI is the most sensitive imaging technique in assessing active MM and is the recommended first-line approach in the United Kingdom despite varied availability.^6^ Diffusion-weighted sequences in conjunction with DIXON fat-suppression techniques increase sensitivity in detecting active lesions and infiltrated marrow and can also discriminate between treated disease.^6^ With the increasing use of sensitive and functional imaging techniques, and greater coverage of the head and neck region, such lesions may be increasingly incidentally detected.

We highlight the extramedullary manifestations of plasma cell tumours in the head and neck region. We categorize these according to their location and share important differentials and diagnostic pitfalls that the radiologist may encounter in this complex anatomical subsite.

## Sinonasal

The sinonasal cavity is the most common site of EMP in the upper aerodigestive tract (38% occur within this subsite).^5^^,^^7^ Their imaging appearances can be non-specific, and targeted biopsy is essential for diagnosis. These are uniformly solid intermediate T2-weighted signal lesions that enhance mildly and restrict diffusion.

### Differential diagnoses

- Lymphoma (Figure 2) is a key differential diagnosis for EMP and can look similar on CT and MRI, namely uniform attenuation/signal and intense restricted diffusion due to their solid composition and hypercellularity. Local and distant lymphadenopathy, which is more common in lymphoma, is a key differentiator.
- Melanoma (Figure 3) is another important differential. A key differentiator is intralesional foci of pre-contrast T1-weighted shortening and T2-weighted hypointensity reflecting the presence of melanin (contains paramagnetic elements) and its propensity to haemorrhage.^8^
- Inverted papilloma (IP) is a benign tumour commonly arising from the lateral wall of the nasal cavity (Figure 4A). Its convoluted cerebriform pattern on T2-weighted imaging is characteristic. IPs are associated with a 9% risk of malignant transformation^9^; therefore, accurate diagnosis and resection of its hyperostotic bony strut are key to complete surgical resection.
- Sinonasal fungal material (Figure 4B) is typically hyperattenuating on CT and can calcify. It owes its classic low T2-weighted signal on MRI to paramagnetic hyphal compounds. Patients with non-invasive fungal disease are often asymptomatic but may experience obstructive symptoms. On the other end of the spectrum, vascular invasion, thrombosis, and resulting tissue ischaemia/necrosis underlie the pathogenesis of invasive and often life-threatening fungal rhinosinusitis.^10^
- Nasal polyps (Figure 4C) are common benign lesions. They appear as symmetrical opacifying lesions in the nasal cavities that are typically hyperintense on T2-weighted imaging with smooth peripheral contrast enhancement.

## Laryngopharynx

**Figure 1.**
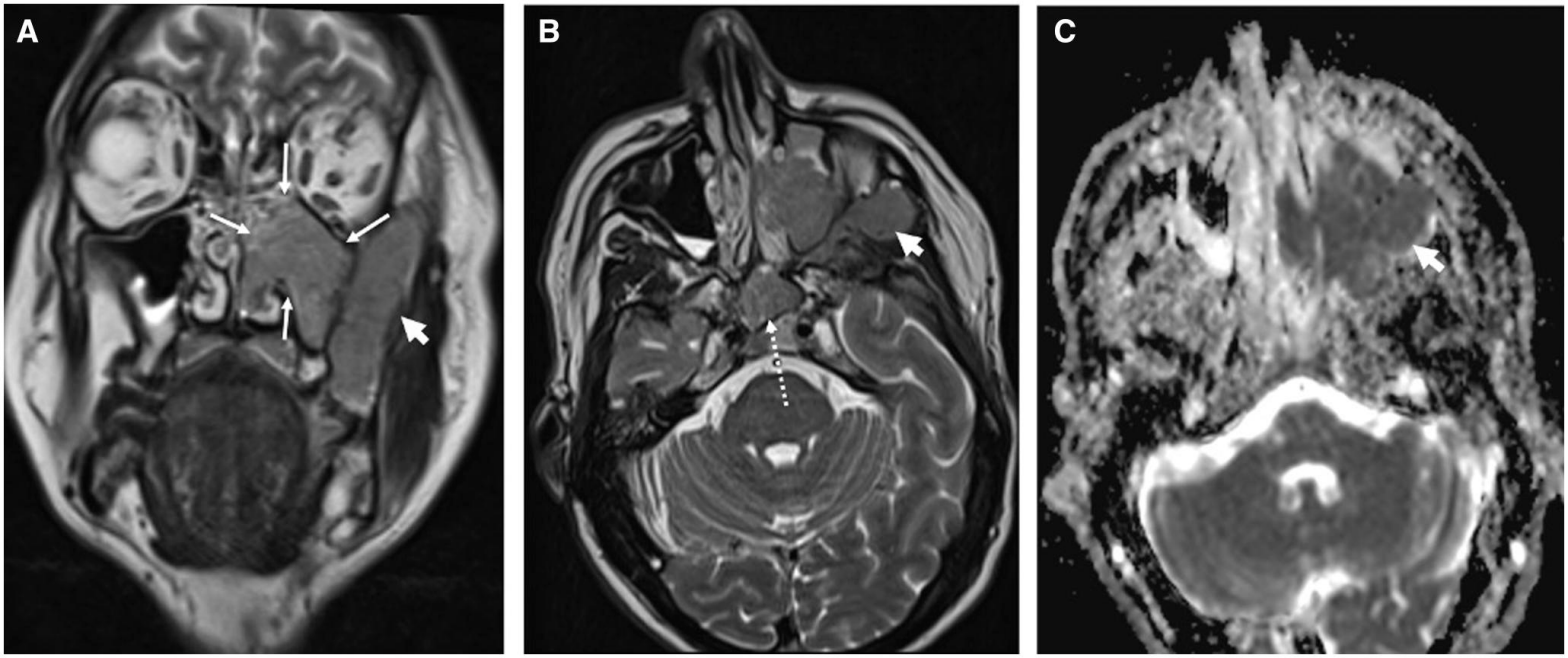
Left sinonasal system EMP (A) and (B) EMP centred on the left maxillary antrum (thin white arrows). The lesion extends into the nasal cavity and via a transosseous route into the bucco-masticator spaces (short white arrow). A component insinuates into the left sphenoid chamber (dotted arrow) in (B). (C) ADC map of the same tumour (short white arrow) demonstrates markedly low signal with the restricted diffusion indicating tumour hypercellularity.

Plasmacytomas arising from ossified laryngeal cartilages are treated as extramedullary entities developing within regions of bony metaplasia rather than within true medullary bone.^11^  Figures 5 and 6 demonstrate well-defined EMPs arising from the thyroid lamina and cricoid ring respectively. Their MRI imaging characteristics are similar to the sinonasal EMP shown in Figure 1.

**Figure 2.**
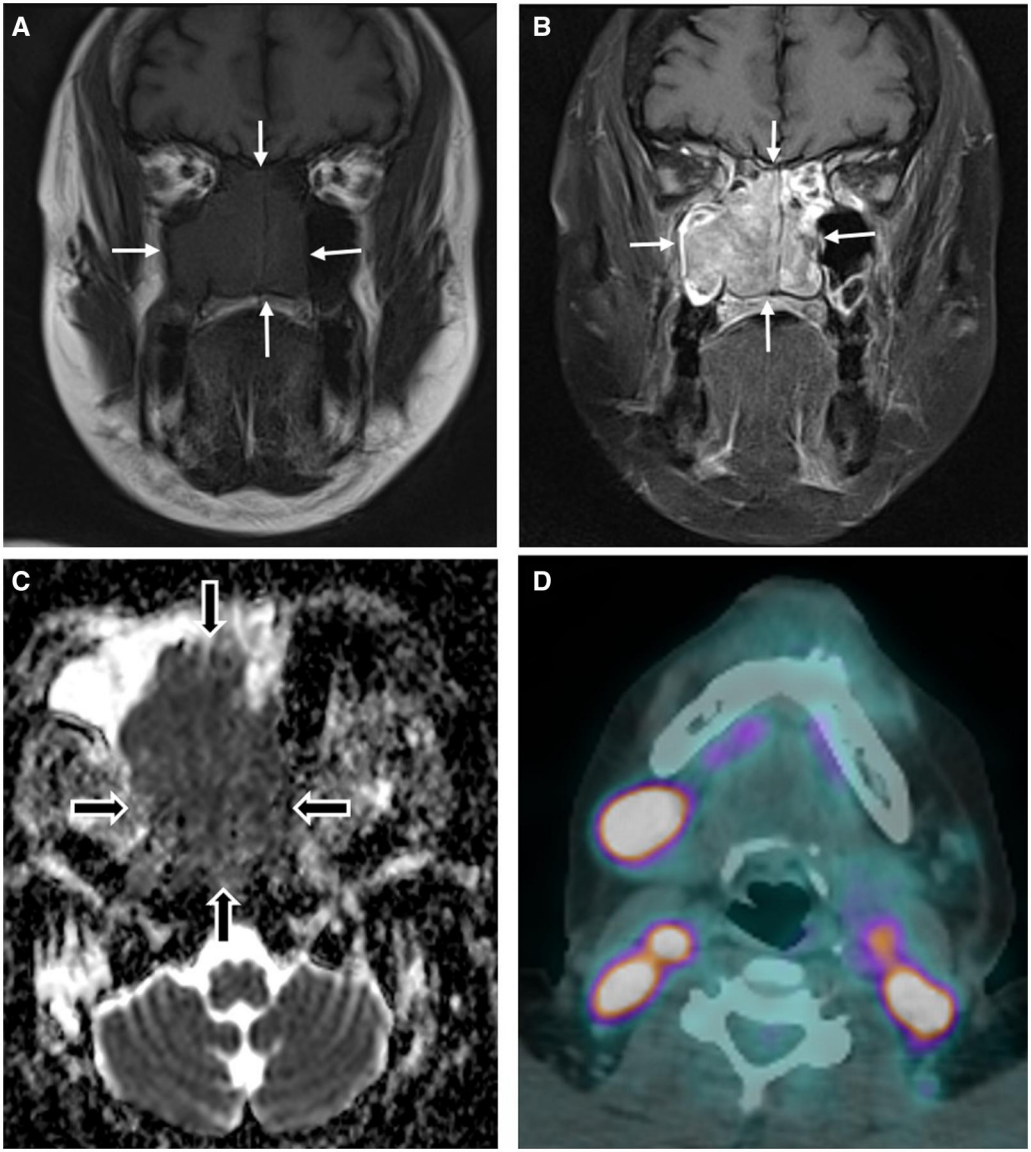
Extranodal NK T-cell lymphoma of the sinonasal system. (A) Coronal non-fat-suppressed T1-weighted and (B) fat-suppressed post-contrast T1-weighted MRI show a low-to-intermediate signal homogeneous nasal cavity and right maxillary antral lesion (white arrows) that enhances relatively homogeneously. (C) ADC map shows markedly low values (black arrows) visually similar to EMP and (D) FDG-PET/CT from the same patient demonstrates multiple avid right level 1b and bilateral level 2 cervical lymphadenopathy which is a useful discriminator from plasma cell neoplasms.

**Figure 3.**
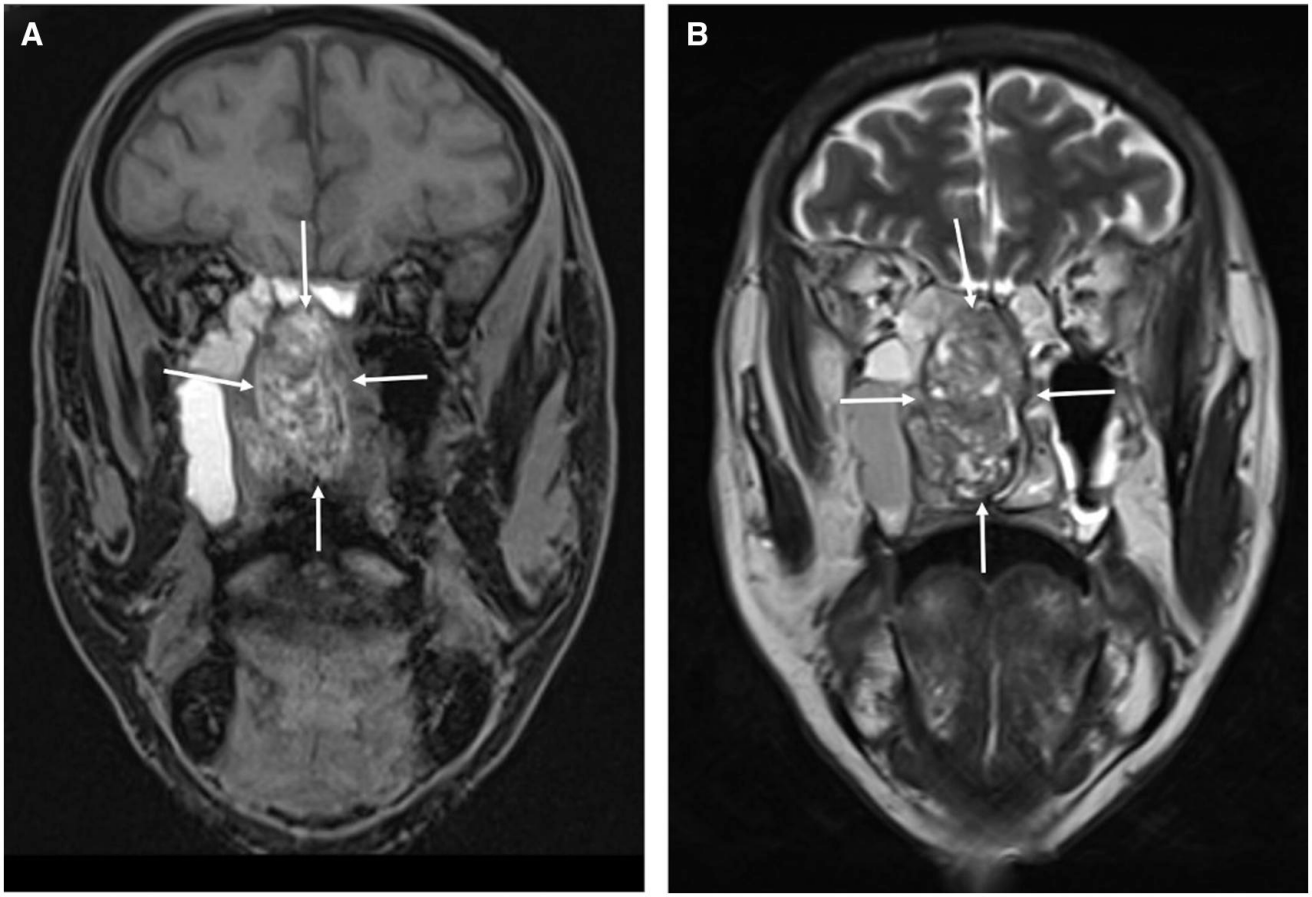
Primary sinonasal melanoma. (A) Coronal fat-saturated pre-contrast T1-weighted MRI and (B) coronal T2-weighted sequence show an expansile heterogeneous nasal cavity lesion (arrows) with foci of intralesional high T1 signal representing melanin/haemorrhage. Foci of high signal and areas of signal void on the T2-weighted sequence (B) are consistent with internal cystic change and old blood products, respectively.

**Figure 4.**
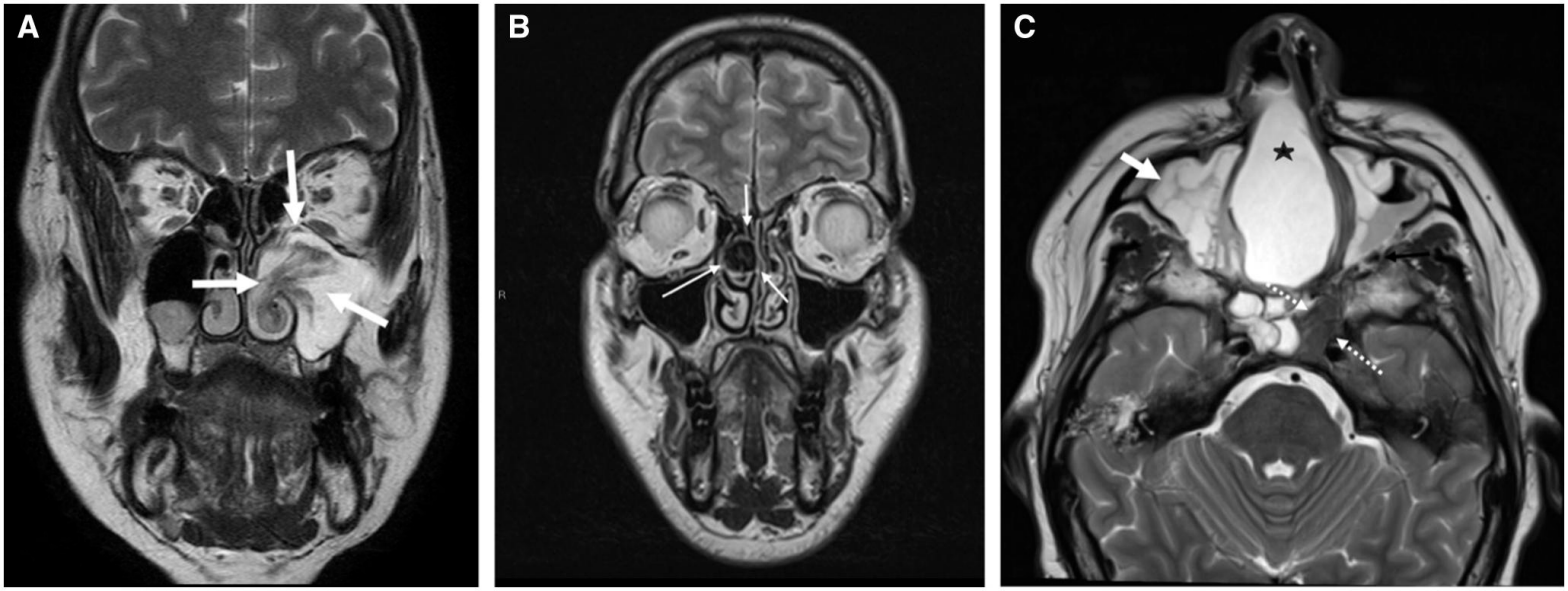
Benign sinonasal pathology. (A) Left nasoantral inverted papilloma: coronal T2-weighted MR image showing the classic cerebriform morphology (white arrows) reflecting its pattern of growth deep to an intact respiratory basement membrane and oedema of the adjacent stroma (type 3 Schneiderian polyp). The lesion insinuates into the maxillary antrum via the native ostium. The posterior accessory ostium can also be a route of IP extension. (B) Fungal ball within a right middle turbinate concha bullosa: coronal T2-weighted MRI showing the classic signal drop-out (thin white arrow) seen with fungal tissue owing to paramagnetic hyphal contents. This will typically show increased (albeit variable) signal on the accompanying T1-weighted sequence; therefore, scrutiny of all sequences is key to avoid confusing this with aerated air cells. There is no erosion of the nasal osteo-cartilaginous structures, but mucocoeles can develop due to obstructing fungal balls. (C) Mega polyp within the nasal cavity: axial T2-weighted MRI shows a large mega polyp filling and expanding the right nasal cavity (star) obstructing the ostiomeatal units. The polyp demonstrates high T2-weighted signal. There is mucosal thickening and submucosal oedema within the maxillary antra (short white arrow) and right sphenoid chamber, and low-signal fungal tissue within the obstructed left sphenoid chamber (dotted white arrow).

**Figure 5.**
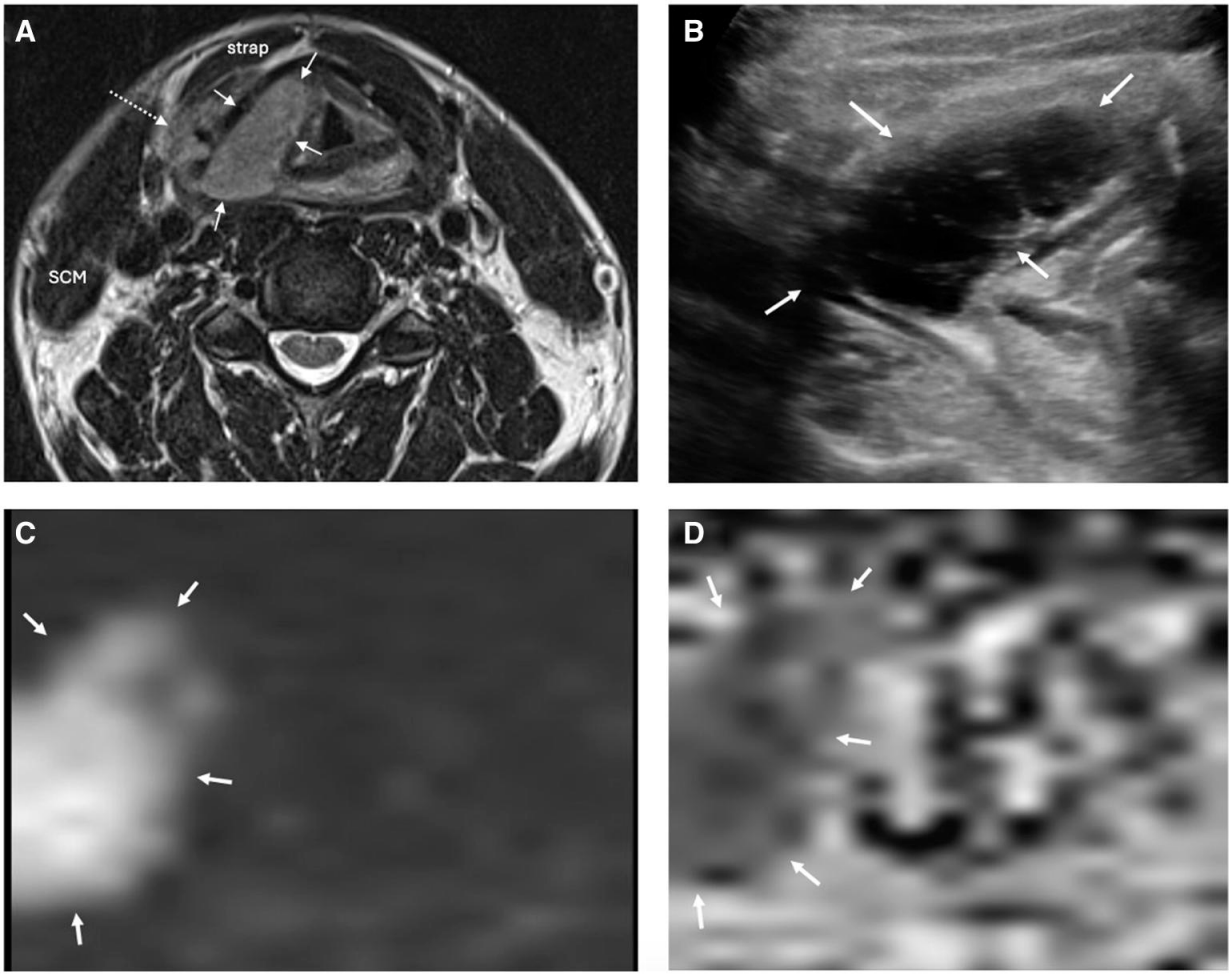
Right thyroid lamina EMP in MM relapse (solid thin white arrows). (A) Axial T2-weighted MRI shows an expansile, intermediate signal mass centred on and eroding the low-signal ossified right thyroid cartilage. There is a superficial component elevating the right strap muscles (dotted arrows) and an endolaryngeal component partially effacing the right paraglottic fat. (B) Transverse high-resolution US image (curvilinear C3-10 MHz probe) shows the hypoechoic mass centred on the thyroid cartilage. Real-time US indicated that the cord was still mobile and facilitated percutaneous image-guided tissue sampling of the superficial component. (C and D) DWI and ADC sequences demonstrating restricted diffusion.

**Figure 6.**
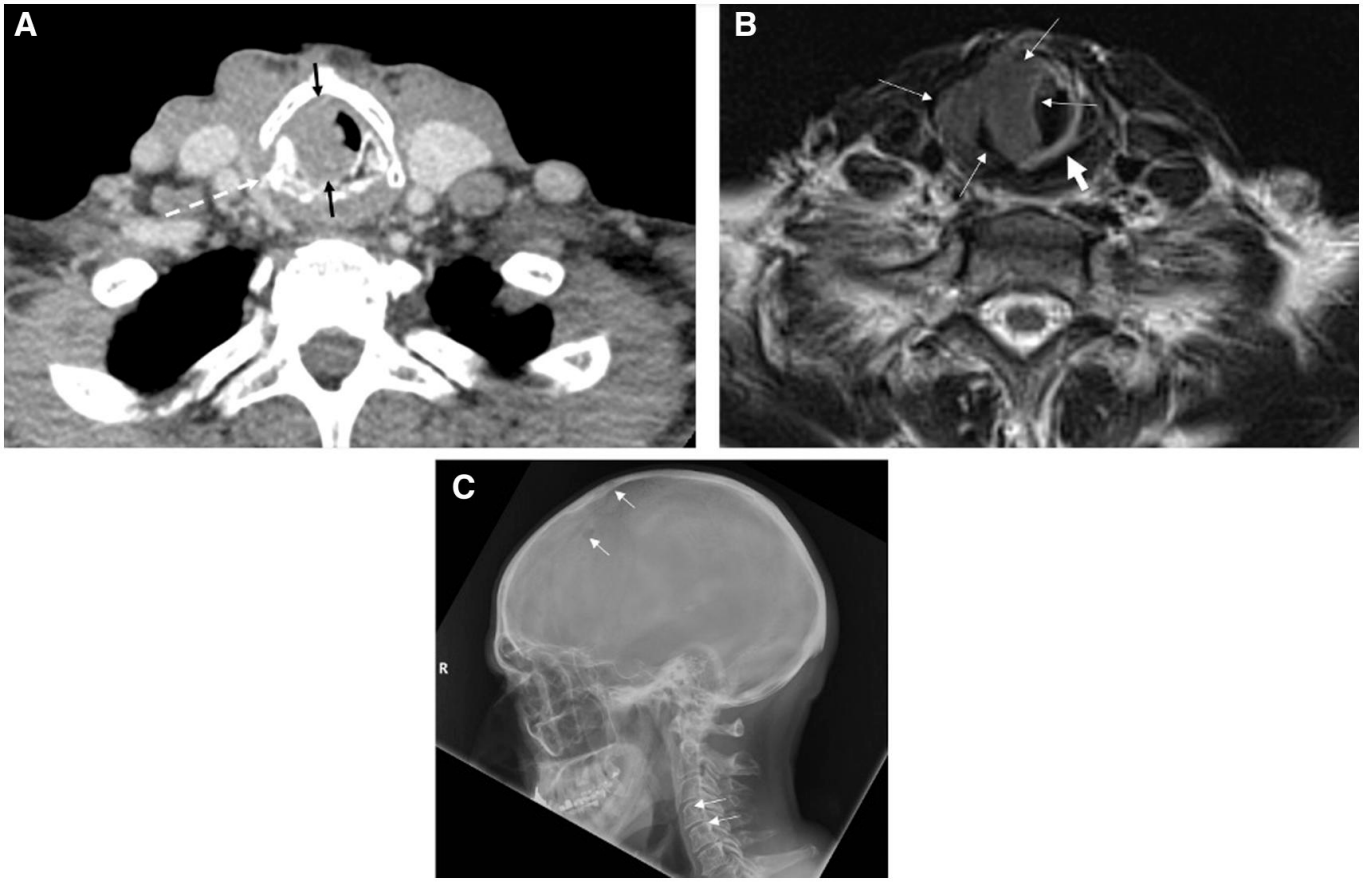
Subglottic and right cricoid ring EMP in MM relapse. (A) Axial CECT shows a homogeneous lobulated mass (thin black arrows) that almost occludes the subglottic larynx. The right cricoid cartilage is sclerotic (dotted arrow). (B) Axial T2-weighted MRI shows homogeneous intermediate signal mass. Note the high T2-weighed signal differentiating tumour (thin white arrow) from normal adjacent laryngeal mucosa (short white arrow). (C) Sagittal radiograph of the calvarium from a historic MM skeletal survey in the same patient as Figure 2A and B demonstrates multiple lytic skull vault and C4-6 vertebral lesions (short thin white arrow).

### Differential diagnoses

**Figure 7.**
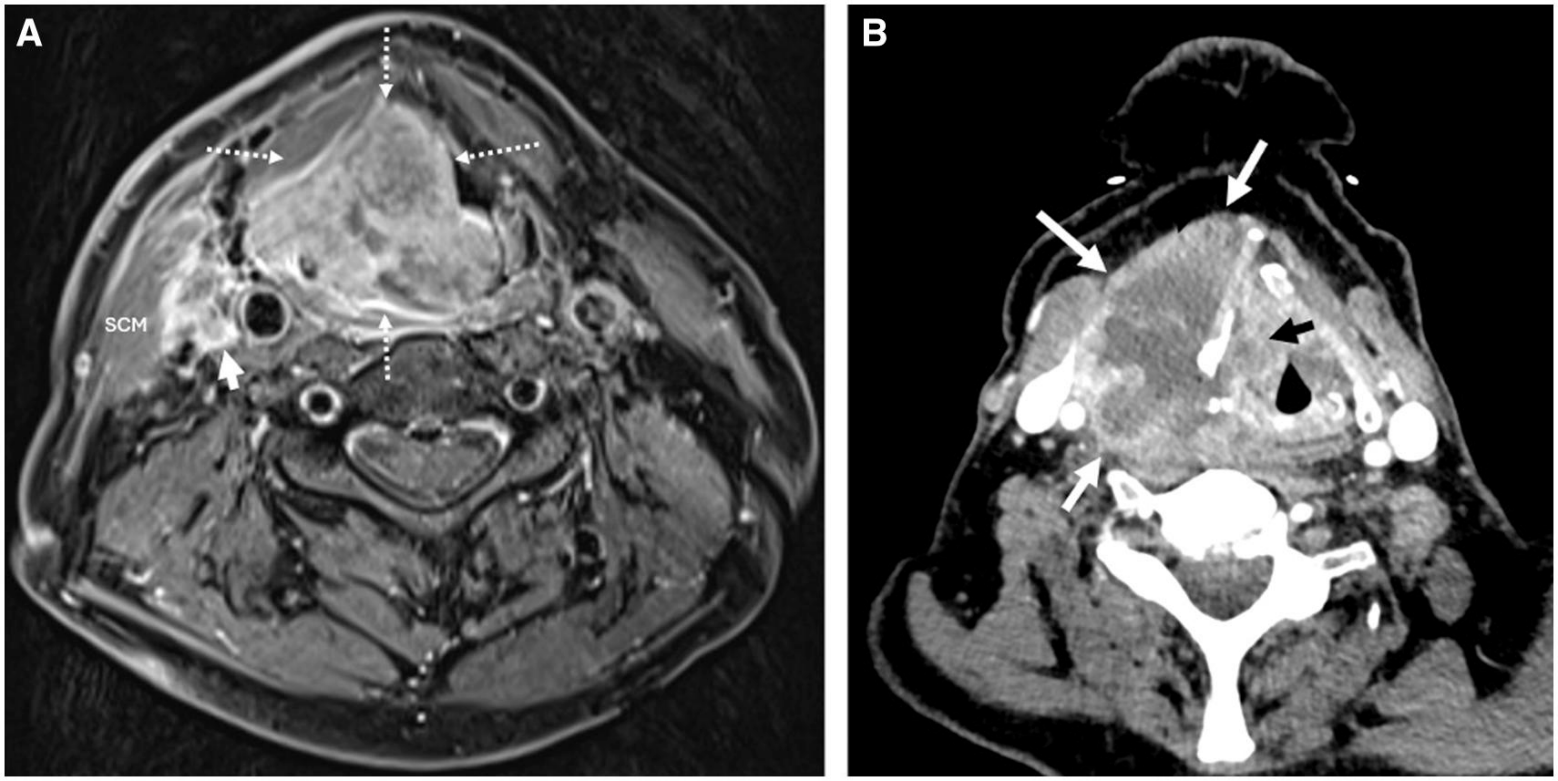
(A) Transglottic SCC: axial post-contrast fat-suppressed T1-weighted MRI shows a bulky right laryngeal tumour (dotted white arrow) with internal necrosis (intralesional areas of non-enhancement) and ipsilateral necrotic level 3 nodal metastasis (short solid white arrow).The metastatic nodes are matted and show radiological features of extracapsular spread into the sternocleidomastoid muscle (SCM). (B) Anaplastic thyroid carcinoma: CECT shows a necrotic (centrally hypoattenuating) mass (solid white arrow) centred of the right lobe of the thyroid gland invading into the larynx (black short arrow).

**Figure 8.**
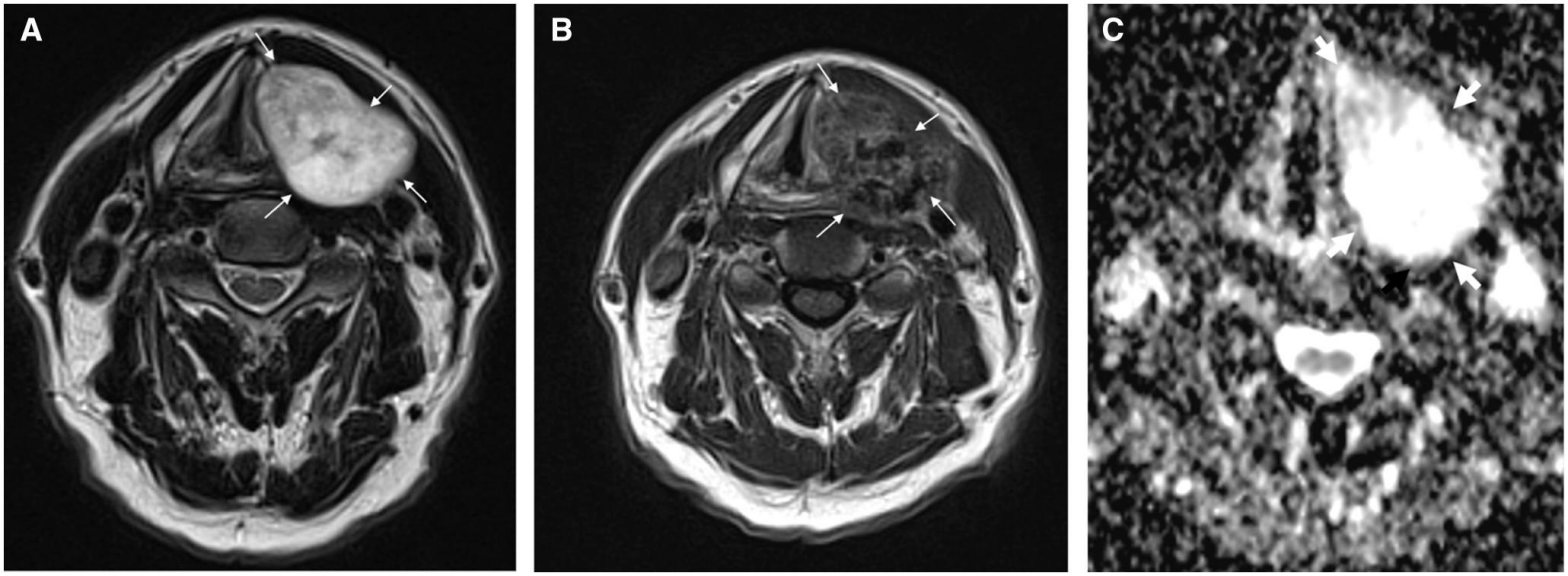
Left thyroid lamina chondrosarcoma. (A) Axial T2-weighted MRI shows a predominantly high signal solid mass replacing the left thyroid cartilage (short thin white arrows). (B) Axial non-fat-suppressed post-contrast T1-weighted MRI demonstrates heterogeneous contrast enhancement. (C) ADC map demonstrates no restricted diffusion.

**Figure 9.**
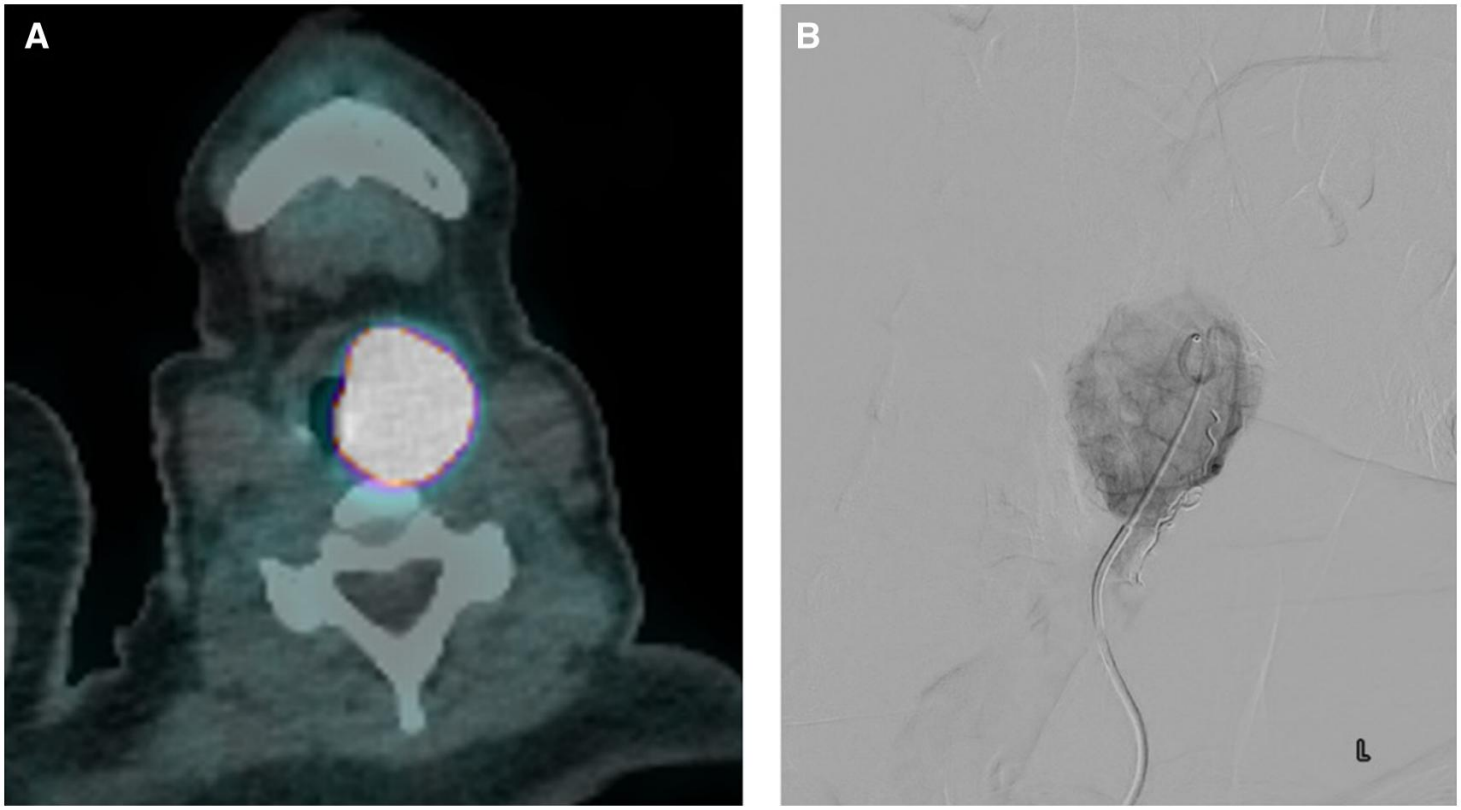
Left supraglottic (aryepiglottic fold) NET. (A) Gallium-68 DOTATATE PET-CT and (B) digital subtraction angiography. MRI appearances were non-specific; however, the intense radiotracer uptake and highly vascular nature of the lesion are more specific for a NET.

- SCCs of the head and neck arise from mucosal surfaces and can be irregular and bulky (trans-spatial). Their intermediate T2-weighted signal and restricted diffusion mimic that of plasma cell neoplasms; however, necrosis, ulceration, and accompanying lymphadenopathy are useful differentiators (Figure 7A).
- Aggressive thyroid malignancies, namely anaplastic carcinoma and thyroid lymphoma, are rare entities (Figure 7B). Early diagnosis is critical as these highly infiltrative tumours rapidly progress with patients often requiring airway support. These can be differentiated from plasmacytomas by identification of thyroid gland rather than the thyroid cartilage as the epicentre of disease and the presence of non-necrotic cervical lymphadenopathy, which may be widespread.
- The markedly elevated T2-weighted signal of chondrosarcomas (greater than that demonstrated with plasmacytomas) is due to the high-water content of its hyaline matrix (Figure 8). Mineralization and/or haemorrhage in chondrosarcomas can result in their classic central “ring and arc” chondroid calcification on CT and heterogeneous punctate enhancement on MRI. These features are not usually identified in EMPs.
- Neuroendocrine tumours (NETs) can demonstrate non-specific CT/MRI appearances. Tumour grade dictates FDG uptake on PET-CT; however, the expression of somatostatin receptors by all NETs and their ability to concentrate somatostatin analogues makes functional imaging using gallium-68 DOTATATE radiotracer a superior problem-solving tool (Figure 9).

## Facial skeleton

**Figure 10.**
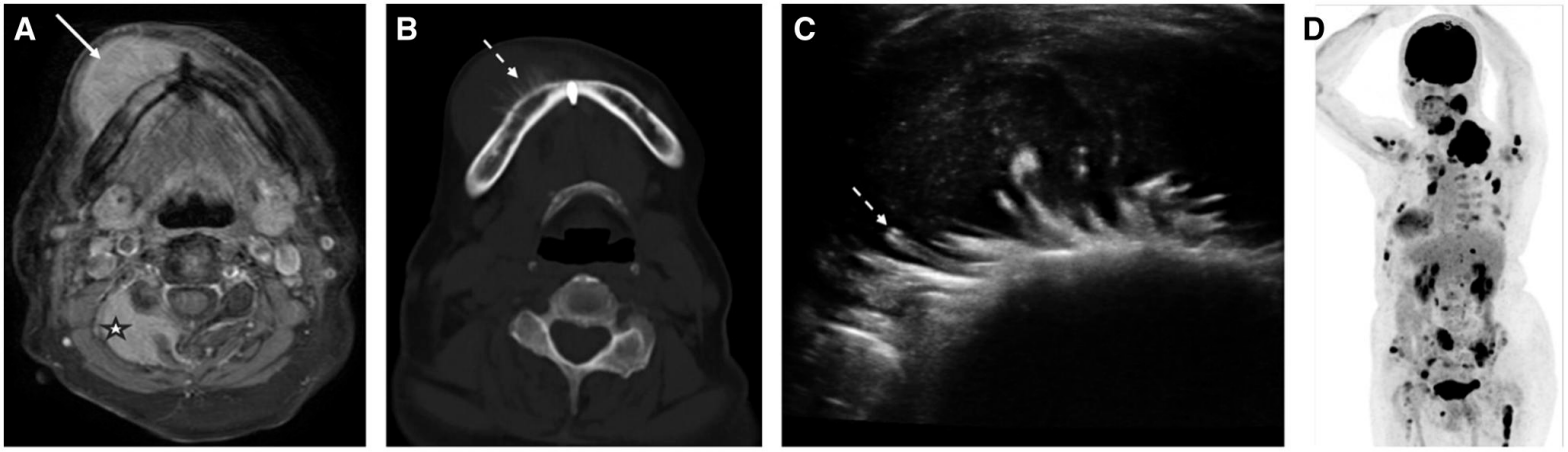
EMP of the right mandible. (A) Axial fat-suppressed post-contrast T1-weighted MRI shows a homogeneously enhancing lesion arising from the infiltrated right mandible (white arrow). There is further EMP infiltrating the right paraspinal muscles and the C3 vertebral facet and lamina (star). (B) and (C) Axial NECT and US show aggressive sunburst periosteal reaction along the buccal cortex of the right mandible consistent with rapid tumour growth (dotted arrow). The lesion is hypoechoic on US. (D) Maximum intensity projection (MIP) from a whole-body FDG-PET-CT in oblique plane demonstrates multiple FDG-avid MM deposits throughout the body.

Extramedullary plasmacytoma can arise from the facial bones and manifests as a highly exophytic soft tissue mass with or without accompanying bony changes (Figure 10).

### Differential diagnoses

**Figure 11.**
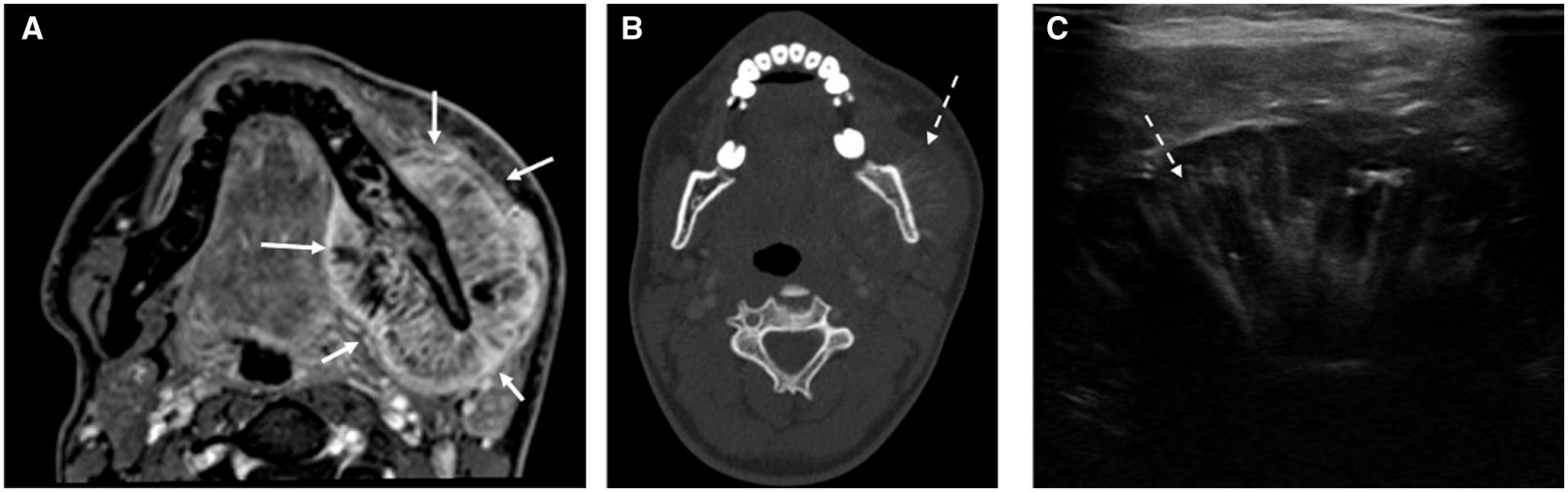
Osteosarcoma of the left mandible. (A) Axial fat-suppressed post-contrast T1-weighted MRI shows an aggressive soft tissue lesion (white arrows) centred on the left mandible that enhances heterogeneously. Intralesional low internal signal reflecting its osteoid matrix, differentiating this from EMPs. (B) Axial CT (bone algorithm) shows aggressive sunburst periosteal reaction along the lingual and buccal cortices of the infiltrated sclerotic left mandible (dotted arrow). (C) US of the left mandible shows heterogeneous soft tissue with linear hyperechoic periosteal reaction. Note the similar appearances of Figures 10B and C and 11B and C, which highlight MRI as a crucial modality in differentiating EMP from osteosarcoma.

- Osteosarcoma of the head and neck is a mimic of EMP in the facial bones (Figure 11). The key differentiator is the presence of a calcified and low MRI signal osteoid matrix in osteosarcomas. Osteosarcoma pulmonary metastasis can be calcified and cause spontaneous pneumothorax.^12^

**Figure 12.**
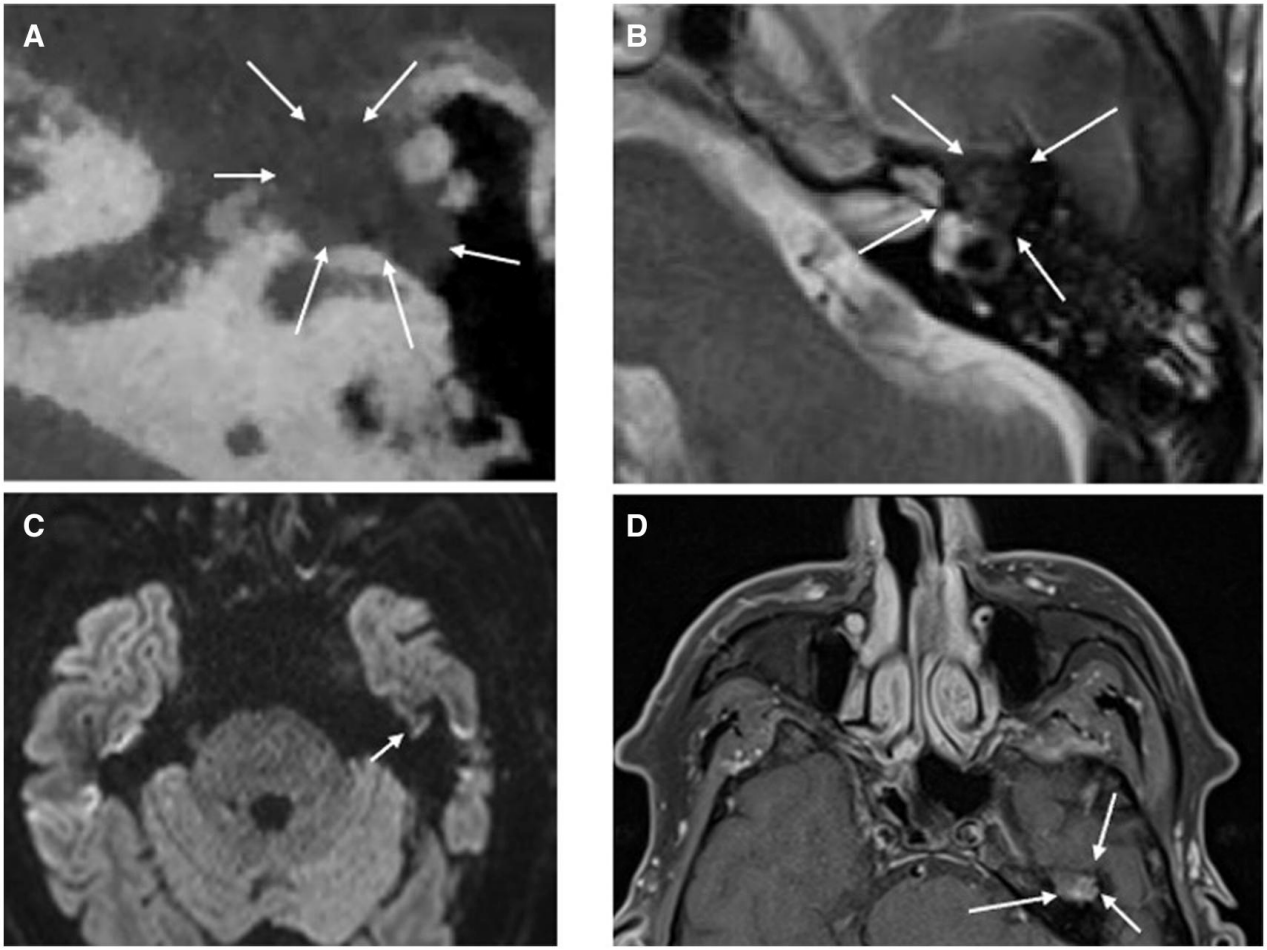
EMP centred on the left geniculate ganglion. (A) Axial non-contrast cone beam CT of the left temporal bone demonstrates an expansile mass (arrows) within the middle ear eroding the otic capsule, geniculate ganglion and tegmen tympani, and laterally displacing the ossicles. (B) Axial T2-weighted MRI shows an intermediate-to-low signal lesion (short white arrow) which restricts on figure (C) and enhances on the (D) fat-saturated post-contrast T1-weighted image (white arrow).

## Skull, skull base, and spine

The bone manifestations of plasma cell neoplasms are well documented in the literature, classically lytic lesions with or without an extraosseous soft tissue component (refer to Figure 8C, which is referred to as “pepper-pot skull” when lesions are numerous). The cross-sectional imaging features of skull base myeloma are less well described. Figure 12 demonstrates EMP centred on and eroding the geniculate ganglion in a patient with dense facial paralysis.

### Differential diagnoses

**Figure 13.**
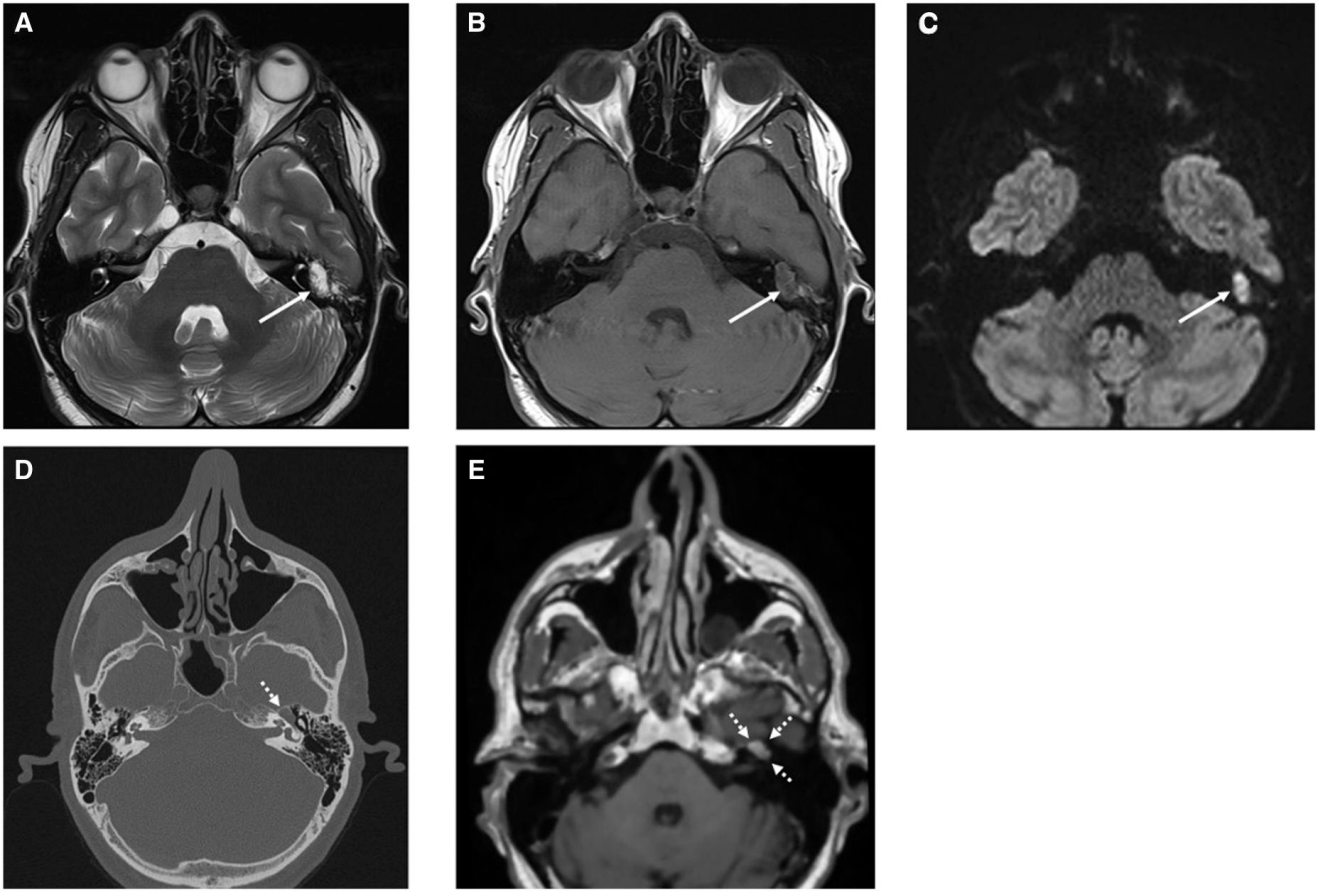
Benign differential diagnoses of the middle ear. (A–C) Acquired cholesteatoma centred on the left mastoid antrum. (A) Axial T2-weighted MRI demonstrates an ovoid and expansile soft tissue lesion filling the antrum. This hyperintense T2-weighted signal (higher than that seen with EMPs) is a key finding as cholesteatomas and EMPs share similar T1-weighted and restriction characteristics, that is, low-intermediate signal on the axial T1-weighted sequence (B) and restricted diffusion (C). In contrast to EMPs, cholesteatomas do not enhance (not shown here). (D, E) Facial nerve schwannoma centred on the left geniculate ganglion. (D) NECT shows lobulated soft tissue (dotted white arrow) expanding the facial nerve canal extending from the labyrinthine segment and involving the geniculate ganglion and anterior portion of the tympanic segment. (E) Non-fat suppressed multiparametric post-contrast T1-weighted MRI demonstrates homogeneous enhancement. There is no restricted diffusion (not shown here).

- Cholesteatoma (Figure 13A–C) is a benign but locally destructive entity that is in essence, keratin-producing squamous epithelium and desquamation debris. It is commonly found in the tympanic cavity following chronic middle ear infections but can also develop within the petrous apex and external auditory canal. The high T2-weighted signal and poor/non enhancement reflecting keratin is a classic feature in conjunction with low T1-weighted signal and restricted diffusion.^13^
- Facial nerve schwannomas (Figure 13D and E) show a predilection for the geniculate ganglion but can involve multiple nerve segments. Lesions typically widen/remodel the canal and demonstrate a fusiform growth pattern although a dumb-bell morphology can also be seen.^14^ They tend to demonstrate homogeneous enhancement, but this can be dependent on size as larger lesions often undergo cystic degeneration; lesions do not restrict.^14^

## Cutaneous, subcutaneous, and muscular structures

**Figure 14.**
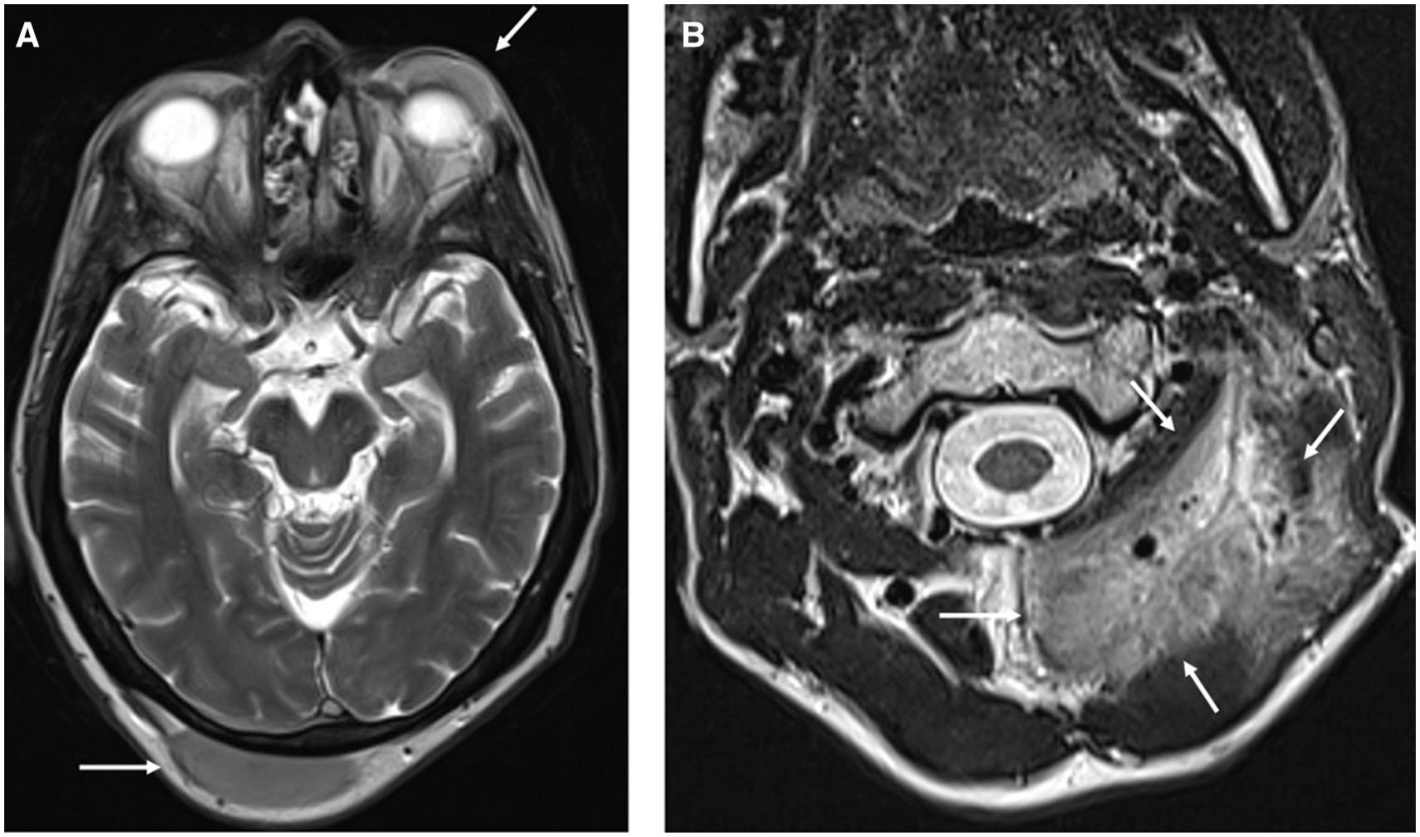
EMPs of the head and facial soft tissues. (A) Axial T2-weighted MRI of the brain show EMPs (white arrows) arising from the right occipital scalp and left upper eyelid (no globe/post-septal involvement). EMP in the perivertebral compartment. (B) Axial T2-weighted MRI shows an intermediate signal lesion infiltrating trapezius and the paraspinal muscles of the left upper neck (arrowed).

A wide range of lesions can arise within these subsites (Figure 14) (ectodermal and mesodermal origin). Many of these are ultimately non-specific on imaging and will require a biopsy for definitive characterization.

### Differential diagnosis

**Figure 15.**
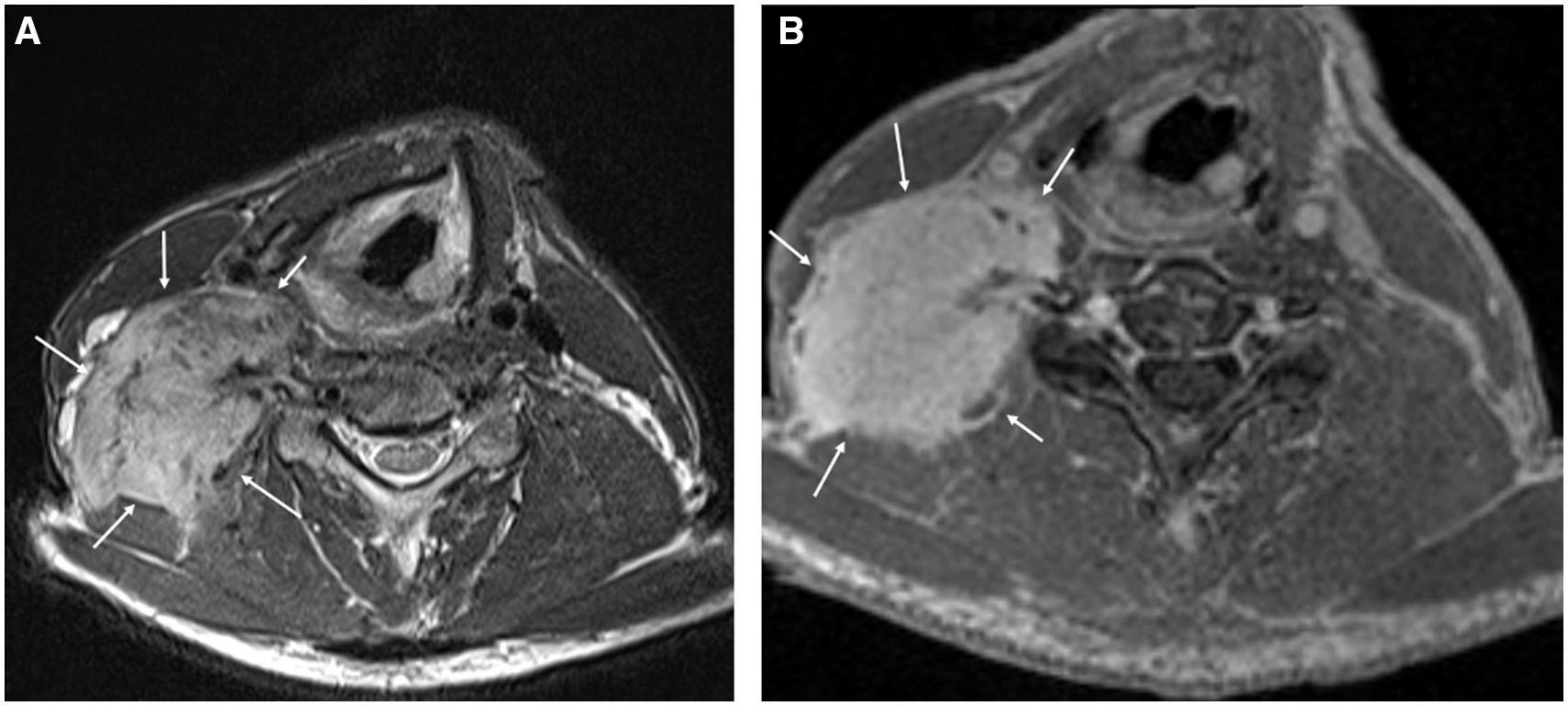
Desmoid fibromatosis. (A) Axial T2-weighted MRI shows an ill-defined lesion infiltrating the right scalene muscles and levator scapulae (solid white arrows). The tumour is predominantly T2-weighted hyperintense; intralesional heterogeneity reflects varying quantities of myofibroblasts, extracellular collagen, and myxoid matrix. Linear low-signal radiating bands correspond to collagen bundles. (B) Non-fat-suppressed post-contrast T1-weighted MRI shows avid tumour enhancement (solid white arrows).

- Desmoid fibromatosis (Figure 15) is a mesenchymal tumour with a predilection for the paediatric and young adult population. Twenty percent of cases occur at sites of previous trauma or surgery. Its intermuscular location and presence of a fascial “tail” are suggestive imaging features.^15^ The locally invasive nature of desmoid fibromatosis can cause airway, vascular, and neural (eg, brachial plexus) compromise; this limits curative surgery and, if operable, correlates with high rates of local recurrence.^15^
- Soft tissue sarcomas should be considered in cases of rapid growth, pain, bulky lipomatous lesions (>5 cm), and/or the presence of internal non-adipose tissue within lipomatous lesions.

## Conclusion

Soft tissue EMPs within the head and neck region, solitary or in the context of MM, can be radiologically challenging entities. We have described the key imaging characteristics, the primary differential diagnoses, and relevant anatomy. Knowledge of these will be helpful to the radiologist, particularly in the context of increasing availability of sensitive whole-body imaging techniques in the assessment of patients with plasma cell disorders.

## Funding

None declared.

## Conflicts of interest

None declared.
